# Rodent Host Abundance and Climate Variability as Predictors of Tickborne Disease Risk 1 Year in Advance

**DOI:** 10.3201/eid2509.190684

**Published:** 2019-09

**Authors:** Emil Tkadlec, Tomáš Václavík, Pavel Široký

**Affiliations:** Palacký University Olomouc, Olomouc, Czech Republic (E. Tkadlec, T. Václavík);; Institute of Vertebrate Biology, Brno, Czech Republic (E. Tkadlec);; UFZ–Helmholtz Centre for Environmental Research, Leipzig, Germany (T. Václavík);; University of Veterinary and Pharmaceutical Sciences Brno, Brno (P. Široký);; Central European Institute of Technology, Brno (P. Široký)

**Keywords:** climate variability, common vole, Ixodes ricinus, Lyme disease, Microtus arvalis, tickborne encephalitis, tickborne diseases, vector-borne infections, Czech Republic, Germany, Austria, Slovenia, Hungary, Slovakia, Poland

## Abstract

Using long-term data on incidences of Lyme disease and tickborne encephalitis, we showed that the dynamics of both diseases in central Europe are predictable from rodent host densities and climate indices. Our approach offers a simple and effective tool to predict a tickborne disease risk 1 year in advance.

In Europe, the generalist tick *Ixodes ricinus* is the principal vector transmitting tickborne pathogens to humans. It has 3 blood-feeding stages, depending on small rodents, such as voles and mice, as the chief reservoir hosts for larval ticks (*1*). The development from larva to nymph is a key aspect in pathogen transmission because the exposure to pathogens is most likely to happen at this stage and therefore directly influences the density of infected nymphs (*2*). Vole population densities change dramatically over time in intervals of 3–5 years (*3*), known as population cycles (*4*). As a result, the chances for questing larvae to encounter a host are expected to vary considerably over time, along with vole population numbers. Investigations of direct relationships between abundance of ticks, disease incidence, and host populations are rare, usually targeting large mammals that provide a blood meal for female adult ticks (*5*). Little is known about the effect of rodent population dynamics on abundance of nymphal ticks (*6*), and studies of the direct effects on disease risk are even rarer (*7*,*8*).

We studied interannual variation in incidences of 2 tickborne diseases (TBDs), Lyme disease (LD) and tick-borne encephalitis (TBE). We aimed to determine if, as suggested by a previous work in North America (*8*), disease risk is related to rodent abundance during the previous year. We also tested the hypothesis that population outbreaks in the common vole (*Microtus arvalis*), along with favorable weather conditions, increase survival of larval ticks and the abundance of nymphal ticks in the following year, thereby resulting in higher disease incidence.

## The Study

**Figure 1:**
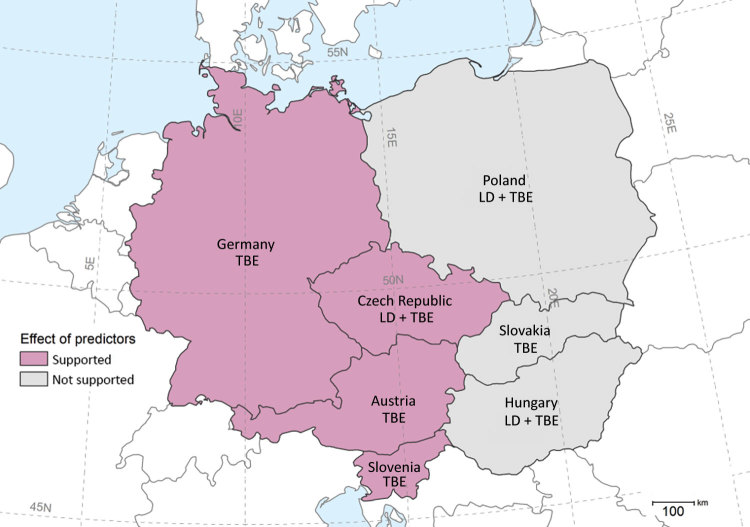
Countries in central Europe where Lyme disease and tickborne encephalitis incidence was analyzed relative to the common vole abundances from the Czech Republic and climate indices, 2000–2017, and where we found evidence for these external predictors. LD, Lyme disease; TBE, tickborne encephalitis.

We analyzed periods of 17–18 years to assess TBD incidences in 7 countries in central Europe (Figure 1; Appendix Figure 1). First, we computed the cross-correlations between disease incidences, vole densities from the Czech Republic, and climate variables to examine the degree of synchrony among their dynamics. Second, we applied autoregressive linear models of order 0–2 to test whether the predictive abilities of vole abundance in year *t* – 1 are supported by data (Appendix). Finally, we tested the influence of climate indices that are known to affect tick ecology (*9*). We used Akaike information criterion for small samples to compare models. The effect included in the model was considered to be strongly supported by data if the model Akaike information criterion was reduced by >2. We obtained data on annual TBD incidences, vole abundance (autumn counts of burrow entrances per hectare), and climate variability (North Atlantic oscillation [NAO] indices) from public databases (Appendix).

**Figure 2:**
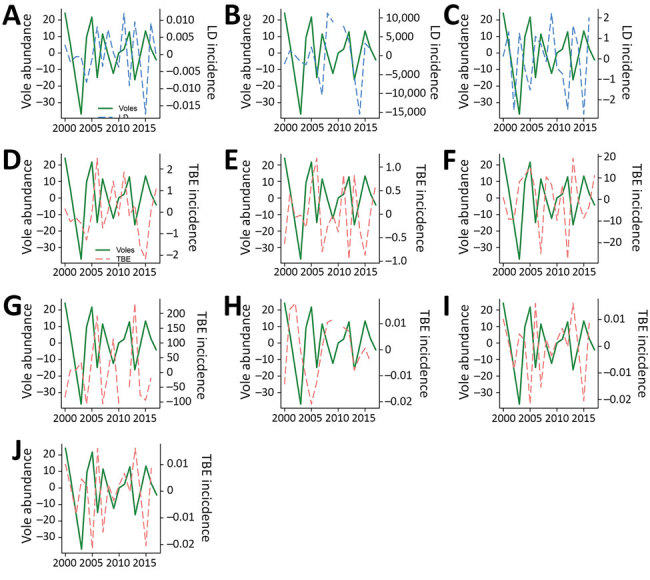
Dynamics of Lyme disease and tick-borne encephalitis incidences in countries of central Europe during 2000–2017 plotted together with the dynamics of common vole abundance (autumn counts of burrow entrances per hectare) in the Czech Republic. Lyme disease incidence in the Czech Republic (A), Hungary (B), and Poland (C); tick-borne encephalitis incidence in the Czech Republic (D), Germany (E), Austria (F), Slovenia (G), Hungary (H), Slovakia (I), and Poland (J). Incidence was plotted together with the dynamics of common vole numbers. Incidences and vole time series were Box–Cox transformed. All variables were detrended by smoothing splines. A data point is missing in the time series of incidence in Hungary. LD, Lyme disease; TBE, tick-borne encephalitis.

LD incidences for 3 countries in central Europe fluctuated over time (Figure 2). Cross-correlation analysis revealed strong positive correlations between incidences in the Czech Republic in year *t* and vole densities in *t* – 1 and negative correlations between the annual NAO index in *t* – 1 (Appendix Figures 2–4). By fitting autoregressive linear models, we found strong evidence that vole abundance in year *t* – 1 and the annual NAO index in *t* – 1 are key to predicting LD incidences during year *t* in the Czech Republic (Table 1); the final model predicted observed incidence with reasonable accuracy (Appendix Figure 5). LD incidence increased with vole densities and decreased with the annual NAO index (Appendix Figure 6).

**Table 1:** Differences in AIC from the best model for Lyme disease incidences as modeled by AR linear models of order 0–2 with vole and abundance annual NAO index as external predictors, 3 countries in central Europe, 2000–2017*

Country and model structure	Order of AR model		
	0	1	2
Czech Republic			
Pure AR model	4.1	3.2	5.0
Voles*_t_*_–1_	2.9	2.6	4.9
NAO annual index*_t_*_–1_	2.8	3.2	3.7
Voles*_t_*_–1_ + NAO annual index*_t_*_–1_	0.0	1.3	2.2
Hungary			
Pure AR model	0.0	3.2	4.6
Voles*_t_*_–1_	0.0	2.1	5.6
NAO annual index*_t_*_–1_	3.3	7.2	6.5
Voles*_t_*_–1_ + NAO annual index*_t_*_–1_	4.1	7.0	12.0
Poland			
Pure AR model	0.4	0.0	1.6
Voles*_t_*_–1_	3.1	3.5	4.8
NAO annual index*_t_*_–2_	0.3	0.1	2.3
Voles*_t_*_–1_ + NAO annual index*_t_*_–2_	3.9	4.5	6.5

AIC, Akaike information criterion; AR, autoregressive; NAO, North Atlantic oscillation.

TBE incidence from 7 countries fluctuated greatly from year to year (Figure 2). Cross-correlations showed that TBE incidence was strongly positively correlated with vole density with a lag of 1 year for the Czech Republic, Germany, and Slovenia (Appendix Figure 7). A lag of 1 year for the effect of the annual NAO index was negatively correlated with TBE incidence in the Czech Republic and Germany, whereas a 2-year lag was positively correlated with TBE incidence in Germany and Austria (Appendix Figure 8). Autoregressive linear models showed that including vole abundance from year *t* – 1 improved fit for the Czech Republic, Germany, and Slovenia (Table 2). Adding the effect of the annual NAO index in *t* – 1 to the model improved the fit in the Czech Republic and Germany. The effect of the annual NAO index in *t* – 2 produced better predictive power for Austria. As a result, the best models for TBE incidence in all 4 countries (Czech Republic, Germany, Slovenia, and Austria) included both host abundance and climate effect. Incidence of both diseases fluctuated over time in close synchrony, as revealed for the Czech Republic (correlation coefficient 0.71) and Poland (correlation coefficient 0.70) (Appendix Figure 9).

**Table 2:** Differences in AIC from the best model for tick-borne encephalitis as modeled by AR linear models of order 0–2 with vole and abundance annual NAO index as external predictors, 7 countries in central Europe, 2000–2017*

Country and model structure	Order of AR model		
	0	1	2
Czech Republic			
Pure AR model	4.5	7.4	10.6
Voles*_t_*_–1_	2.8	6.0	10.1
NAO annual index*_t_*_–1_	3.4	6.8	8.1
Voles*_t_*_–1_ + NAO annual index*_t_*_–1_	0.0	3.5	7.5

Germany			
Pure AR model	6.0	7.2	9.2
Voles*_t_*_–1_	3.6	6.4	10.1
NAO annual index*_t_*_–1_	4.8	7.6	9.7
Voles*_t_*_–1_ + NAO annual index*_t_*_–1_	0.0	3.8	7.1

Austria			
Pure AR model	5.3	6.5	9.1
Voles*_t_*_–2_	5.8	7.3	9.3
NAO annual index*_t_*_–2_	5.8	4.1	7.0
Voles*_t_*_–2_ + NAO annual index*_t_*_–2_	0.0	3.5	5.7

Slovenia			
Pure AR model	3.8	5.2	5.7
Voles*_t–_*_1_	0.2	3.3	5.8
NAO annual index*_t–_*_1_	5.2	5.9	3.7
Voles*_t_*_–1_ + NAO annual index*_t_*_–1_	0.0	3.2	4.2

Hungary			
Pure AR model	0.0	3.2	4.6
Voles*_t_*_–2_	0.0	2.1	5.6
NAO annual index*_t–_*_1_	3.3	7.2	6.5
Voles*_t_*_–2_ + NAO annual index*_t_*_–1_	4.1	7.0	12.0

Slovakia			
Pure AR model	1.2	0.0	3.0
Voles*_t_*_–1_	3.5	3.4	7.1
NAO annual index*_t–_*_1_	3.3	1.3	3.0
Voles*_t_*_–1_ + NAO annual index*_t_*_–1_	6.1	5.4	8.1

Poland			
Pure AR model	0.0	1.7	2.0
Voles*_t_*_–1_	2.8	4.2	5.8
NAO annual index*_t_*_–2_	2.3	4.2	6.3
Voles*_t_*_–1_ + NAO annual index*_t_*_–2_	5.2	6.7	8.2

*AIC, Akaike information criterion; AR, autoregressive; NAO, North Atlantic oscillation.

## Conclusions

For 4 of the 7 countries in Europe we studied, our results show support for the hypothesis that incidence of 2 TBDs should lag 1 year behind the rodent host density because of the beneficial effect on survival of *I. ricinus* larvae (*10*). Our results agree with evidence from North America that the number of *I. scapularis* nymphs can be predicted by small rodent density from the preceding year (*7*,*8*). In addition, acorn abundance was demonstrated to predict the nymph densities equally well 2 years ahead (*7*,*8*). Hence, results from North America indicated a complete causal mechanism for variation in LD incidences over time, starting with abundant acorns in year *t* – 2, which increased the population of rodents in year *t –* 1. High rodent density then led to the increased number of nymphs in year *t*, resulting in a greater disease incidence in humans. Our data support this mechanism and suggest that the LD system in North America based on *I. scapularis* and that in Europe based on *I. ricinus* might be functionally quite similar, differing primarily in the species involved, and that this mechanism also applies to other tickborne diseases, such as TBE.

Unlike bank voles (*Myodes glareolus*) and *Apodemus* mice (*2*), the common vole occupying open farmland habitats has never been regarded as the chief host for *Ixodes* larvae in central Europe, though it is well-known as a competent host for pathogens and larval ticks (*11*,*12*). We suggest 2 explanations for the role of this rodent in disease transmission. First, common voles can be encountered frequently in forests or wetlands in peak years (*13*), when their densities often exceed 2,000 voles/hectare. Thus, the common vole can act as an amplifying host and contribute substantially to the whole population of suitable hosts for larval ticks. The voles’ irruptive population dynamics can readily explain the upsurge in TBE disease prevalence observed in the Czech Republic in 2006, which occurred after a massive population outbreak of voles during 2004–2005 (*14*). Second, population fluctuations of most rodent species are spatially synchronized across large geographic areas (*15*). Therefore, common vole abundance is a correlative measure for bank voles or mice.

Our observation that the annual NAO index in *t* – 1 was able to improve model fit is in agreement with known tick ecology. A negative annual NAO index is generally associated with cold, snowy winters and moderate summers that are wetter, which can help larval ticks conserve body water and thus increase their survival to the nymphal stage (*9*). The positive effect of the annual NAO index in *t* – 2 might signify a generally warmer year that can be related to mast seeding of trees, triggering the growth of rodent populations.

Some countries in central Europe, such as the Czech Republic, Germany, and Poland, have built programs to monitor common vole densities. These data are stored in public electronic databases and thus can be used readily for predicting TBDs, using the methods we describe.

Appendix Additional information regarding rodent host abundance and climate variability as predictors of tickborne disease risk 1 year in advance.
