# Supplementary material for: Rodent Host Abundance and Climate Variability as Predictors of Tickborne Disease Risk 1 Year in Advance
Source: Emerg Infect Dis. 2019 Sep;25(9):1738–41. doi: 10.3201/eid2509.190684 (PMC6711232; doi:10.3201/eid2509.190684)
Supplement: Appendix — Additional information regarding rodent host abundance and climate variability as predictors of tickborne disease risk 1 year in advance. [file 19-0684-Techapp-s1.pdf]

Article DOI: https://doi.org/10.3201/eid2509.190684

Rodent Host Abundance and Climate Variability as Predictors of Tickborne Disease Risk 1 Year in Advance

Appendix

Material and Methods

Tickborne disease data. Annual LD and TBE incidences in the Czech Republic between 2000 and 2017 were obtained from the Information System for Infectious Diseases (formerly Epidat) managed by the National Institute of Public Health. The data on annual incidences for the other six central European countries were obtained from the Global Infectious Diseases and Epidemiology Online Network (GIDEON, https://www.gideononline.com/product-resources/?app). As length of data is critical in time series analyses, we discarded time series shorter than 15 years. Consequently, we studied LD incidences for the Czech Republic, Slovakia and Poland, and the TBE incidences for the Czech Republic, Germany, Austria, Slovenia, Hungary, Slovakia and Poland (Figure 1, https://wwwnc.cdc.gov/EID/article/25/9/19-0684-F1.htm).

Vole data. Common vole abundances in the Czech Republic from 2000 to 2017 were obtained from the Central Institute for Supervising and Testing in Agriculture which estimates population densities twice a year during the spring (March–April) and autumn (October–November) by counting the number of active burrow entrances per hectare in crop fields. Active openings are indicated by the smooth margins of the entrances, fresh plants placed inside the burrow openings, fresh heaps of soil and/or fresh droppings. In each of the 77 districts, an administrative unit of on average 1000 km^2 in size, ≈10 sites are surveyed by counting burrow entrances while walking along four 100-m strips, each 2.5 m wide. The counts collected across a total area of 1000 m^2 are then multiplied by 10 to obtain the numbers per hectare. The indices for all districts were averaged to obtain the vole index for the whole country. We used the indices for autumn vole populations collected in fodder as a proxy for annual density estimates. Autumnal

densities exhibit much higher yearly variation than spring densities and are therefore commonly used in small rodent dynamics studies (*1*). This index of vole population density in the Czech Republic was used as a general predictor of disease incidences in all neighboring countries.

Climate data. We included two indices of large-scale climate variability, which are often more effective predictors of population behavior in animals than single weather variables (*2*). Specifically, we applied station-based Annual NAO index and station-based DJFM NAO index (also known as winter NAO index). Whereas the former simultaneously captures the effects of both temperature and precipitation throughout the year, the latter measures the prevailing character of winter, the harshest period of year for most organisms in the seasonal environment of central Europe. Because the annual NAO index outperformed the DJFM NAO index, we show results only for the former. The indices are available from https://climatedataguide.ucar.edu/climate-data/hurrell-north-atlantic-oscillation-nao-index-station-based.

Statistical analysis. Most statistical tools for time series were specifically developed for description of time-varying processes. They assume constant mean and variance (stationarity). Though annually aggregated, the disease incidences are counts following Poisson distribution. Temporal trends in incidences are pervasive. Consequently, before performing time series analysis, we stabilized the variance of incidences and vole abundance by Box–Cox transformation (data transformation did not change the major results) with the transformation parameter lambda being also estimated from the data. Then we removed trends in all variables using smoothing splines (function smooth.spline), the common method used to smooth a volatile time series (Appendix Figure 1). The smoothness parameter is estimated directly from data using a generalized cross-validation method, preventing overfitting. We assessed the overall synchrony among dynamics by computing cross-correlations to get insight into the relationship between the incidences in different countries and external variables, such voles and climate variables. To account for the effect of serial autocorrelations among the adjacent incidence values, we applied autoregressive linear models of order 0 to 2 as implemented in R using the function arima (*3*) with vole abundances and climate variable as external predictors

$$X_t = a_0 + a_1 X_{t-1} + a_2 X_{t-2} + a_3 V_{t-d} + a_4 C_{t-d} + \varepsilon_t$$

where X_t are yearly disease incidences, a_0 is an intercept, a_1 to a_2 are autoregressive coefficients measuring the strength of dependence on the previous values, a_3 is a regression

coefficient measuring the strength of a vole effect, $V_{t-\mathrm{d}}$ is autumn Czech vole abundance in year $t - 1$ or $t - 2$, d is a delay of 1 to 2 years, a_4 is a regression coefficient measuring the strength of a climate effect, $C_{t-\mathrm{d}}$ is a climate variable in year $t - 1$ or $t - 2$, and ε_t is a Gaussian noise term quantifying the stochastic variation with a constant mean and variance. The delay $d = 2$ for vole abundances was chosen only for Austria and Hungary, i.e., that is countries with asynchronous incidence dynamics relative to the Czech ones. For climate variables, we were guided by cross-correlations. We avoided building models with more than one climate effect to maximize model parsimony. We used AIC_c for small samples to compare models. The effect included in the model was considered as strongly supported by data if the model AIC_c was reduced by more than 2 (*4*). Finally, we visualized vole and climate effects on disease incidences using generalized additive models which can accommodate nonlinearity in a relationship between two variables.

Results of time-series analysis. Cross-correlation analysis showed that Czech and Polish LD incidence dynamics were synchronous (Appendix Figure 2). A lag of 1 year was observed for the effect of vole densities on LD incidence in the Czech Republic while a lag of 2 years was indicated for Hungary (Appendix Figure 3). A 1-year lag was also identified for the annual NAO index in the Czech Republic, where it was negatively correlated with LD incidences, whereas no pattern or a lag of 2 years was observed for Hungary and Poland, respectively. In the Czech Republic, the negative annual NAO index is associated with a lower annual mean temperature and higher annual precipitation sum (Appendix Figure 4). By fitting autoregressive linear models of order 0–2, we found strong evidence that vole abundance in year $t - 1$ is key to predicting LD incidences during year t in the Czech Republic (Table 1). No effect of vole abundance was found for Hungary and Poland. Including the annual NAO index improved the model fit in the Czech Republic, with the final model predicting observed incidences with a reasonable accuracy (Appendix Figure 5). LD incidences increased with vole densities and decreased with annual NAO index (Appendix Figure 6).

TBE incidence dynamics were synchronous across countries, with the exception of Austria and Hungary (Appendix Figure 2). TBE incidences were strongly positively correlated with vole densities with a lag of 1 year for the Czech Republic, Germany and Slovenia (Appendix Figure 7). A 2-year lag was weakly indicated for Austria and Hungary. A lag of 1 year for the effect of annual NAO index was negatively correlated with TBE incidences in the Czech Republic and Germany, while a 2-year lag was positively correlated with TBE incidences

in Germany and Austria (Appendix Figure 8). Incidences of both diseases fluctuated over time in a great synchrony as revealed for the Czech Republic and Poland, with correlation coefficients of 0.71 and 0.70, respectively (Appendix Figure 9).

References

1. Tkadlec E, Stenseth NC. A new geographical gradient in vole population dynamics. Proc Biol Sci. 2001;268:1547–52. PubMed http://dx.doi.org/10.1098/rspb.2001.1694
2. Stenseth NC, Mysterud A. Weather packages: finding the right scale and composition of climate in ecology. J Anim Ecol. 2005;74:1195–8. http://dx.doi.org/10.1111/j.1365-2656.2005.01005.x
3. R Core Team. R: A language and environment for statistical computing. Vienna: R Foundation for Statistical Computing; 2018.
4. Burnham KP, Anderson DR. Model selection and multimodel inference: a practical information theoretic approach. New York: Springer; 2002.

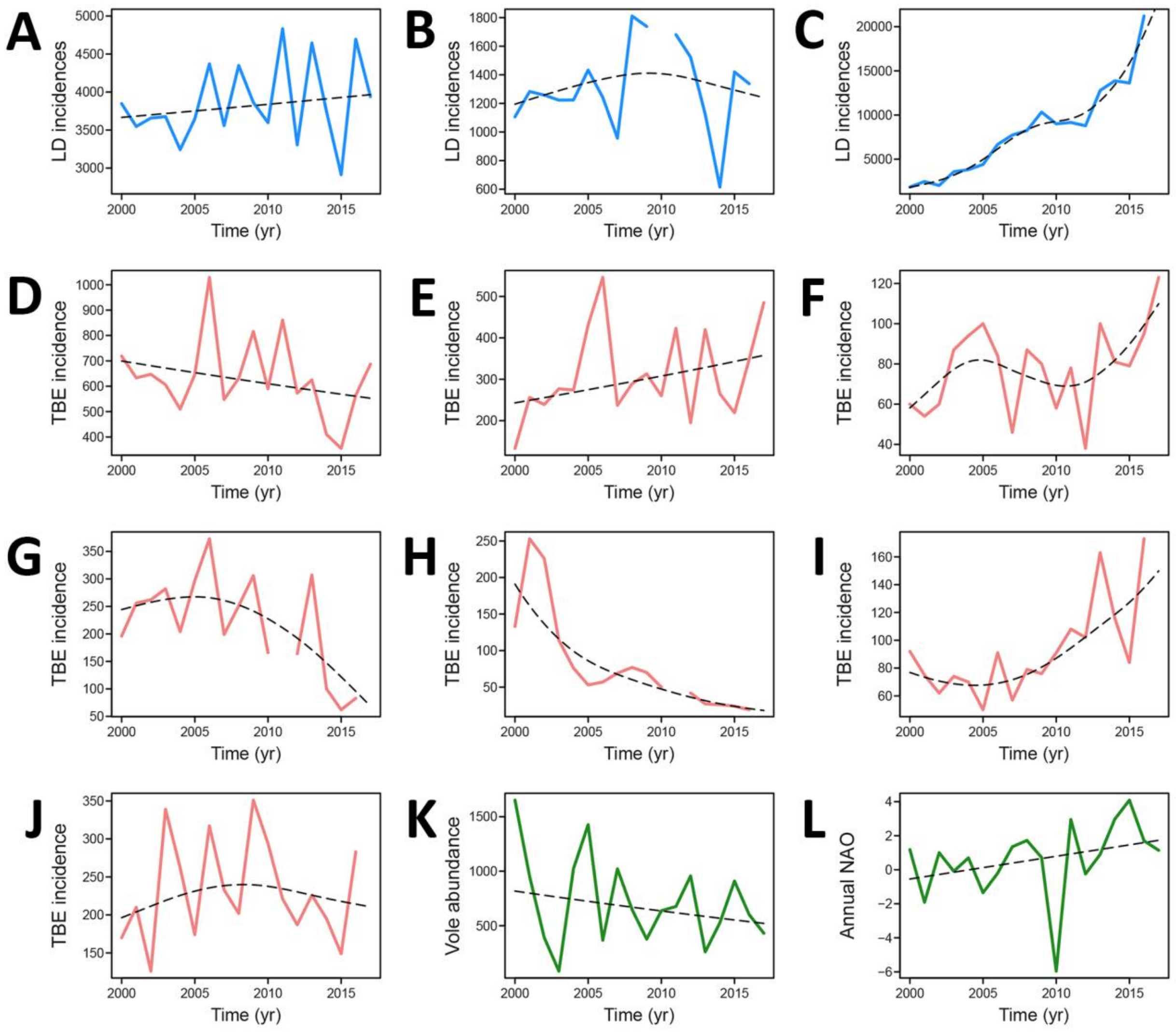

Appendix Figure 1. Trends in original time series (dashed line) of disease incidences (solid line), vole abundance and annual NAO index as modeled by smoothing splines (dashed line). The spar parameter controlling the degree of smoothness was set using a generalized cross-validation criterion. Lyme disease (LD) incidences (blue line) were available for the Czech Republic (A), Hungary (B) and Poland (C). Tickborne encephalitis (TBE) incidences (red line) were available for the Czech Republic (D), Germany (e), Austria (f), Slovenia (G), Hungary (H), Slovakia (I) and Poland (J). Lyme disease incidences in Poland exhibited a steep upward trend. Tickborne encephalitis incidences in Hungary exhibited a steep downward trend. There is a missing data point in the Polish Lyme disease incidences and in the Slovenian and Hungarian tickborne encephalitis incidences.

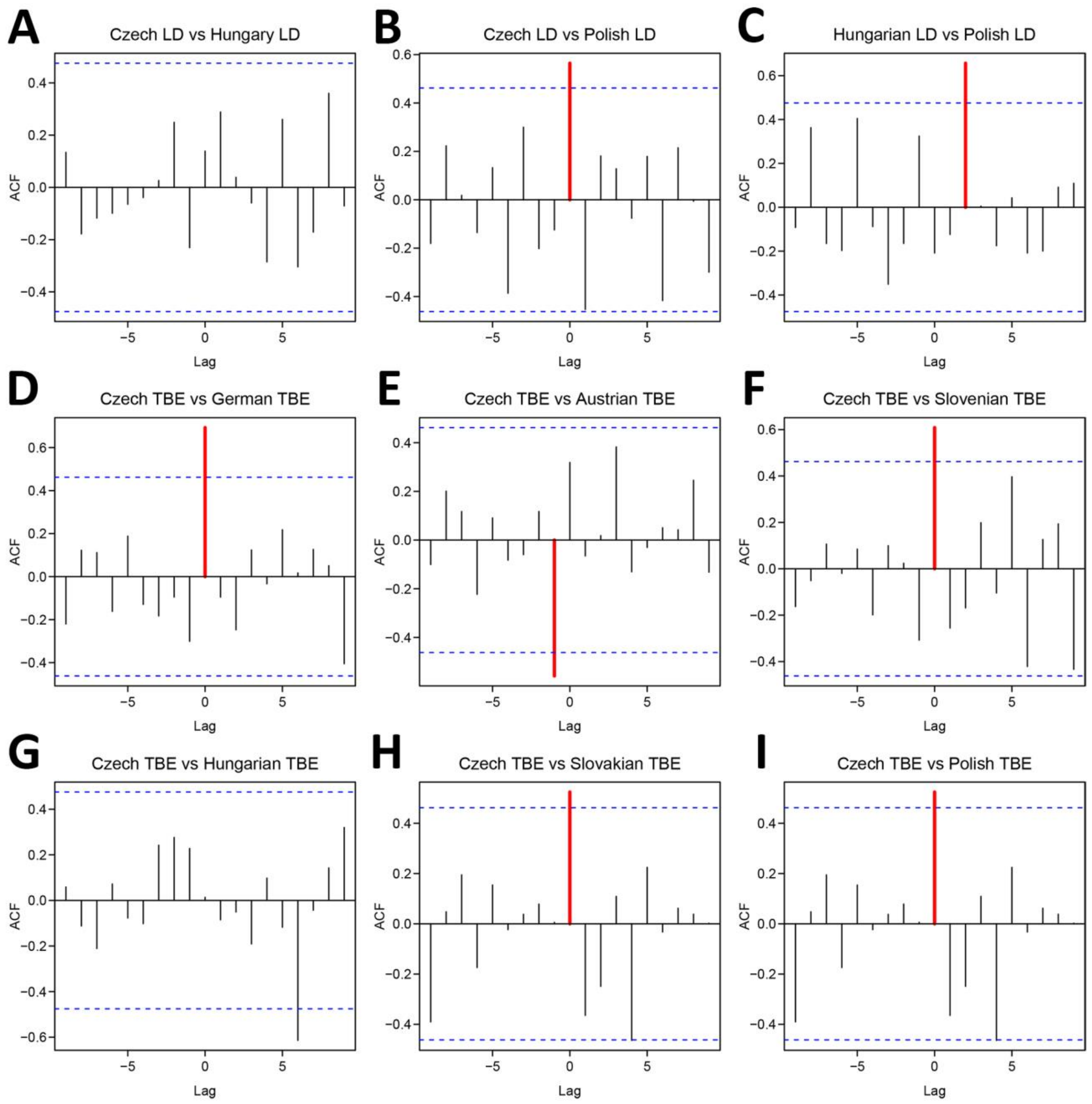

Appendix Figure 2. Cross-correlations showing the degree of temporal synchrony between the incidences from the Czech Republic and other European countries for Lyme disease (LD, A–C) and tickborne encephalitis (TBE, D–I). For Lyme disease, we found synchrony between Czech and Polish incidences. For tickborne encephalitis, Czech incidences fluctuated in synchrony with those from Germany, Slovenia, Slovakia and Poland. The dynamics of Austrian and Hungarian TBE incidences were asynchronous. The meaningful lags are in red.

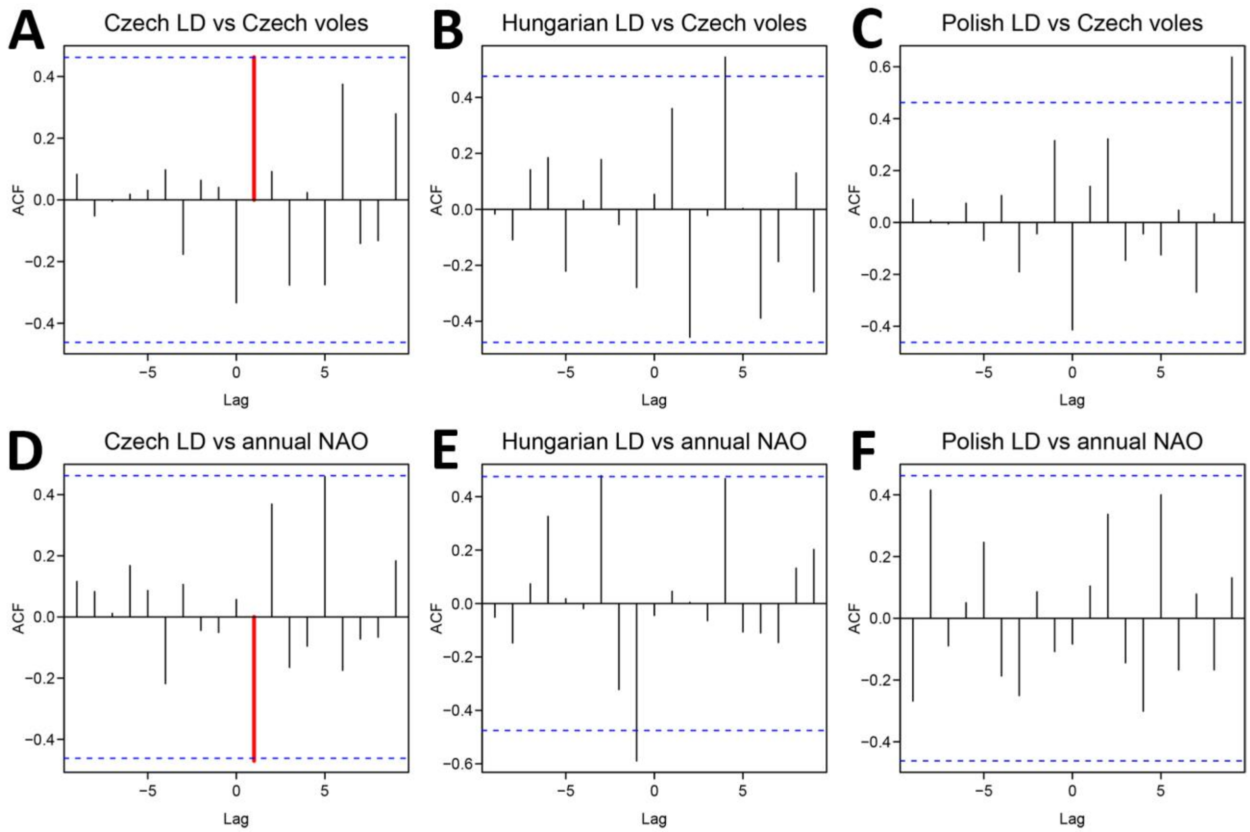

Appendix Figure 3. Cross-correlations between Lyme disease (LD) incidences and Czech voles (A–C) and between Lyme disease incidences and annual NAO index (D–F). The incidences lagged 1 year behind the vole densities in the Czech Republic whereas a lag of 2 years is indicated for Hungary and weakly for Poland. The incidences in the Czech Republic were either negatively correlated with the annual NAO index with a lag of 1 year or positively correlated with a lag of 2 years. A lag of 2 years weakly dominates in Poland. The meaningful lags are in red.

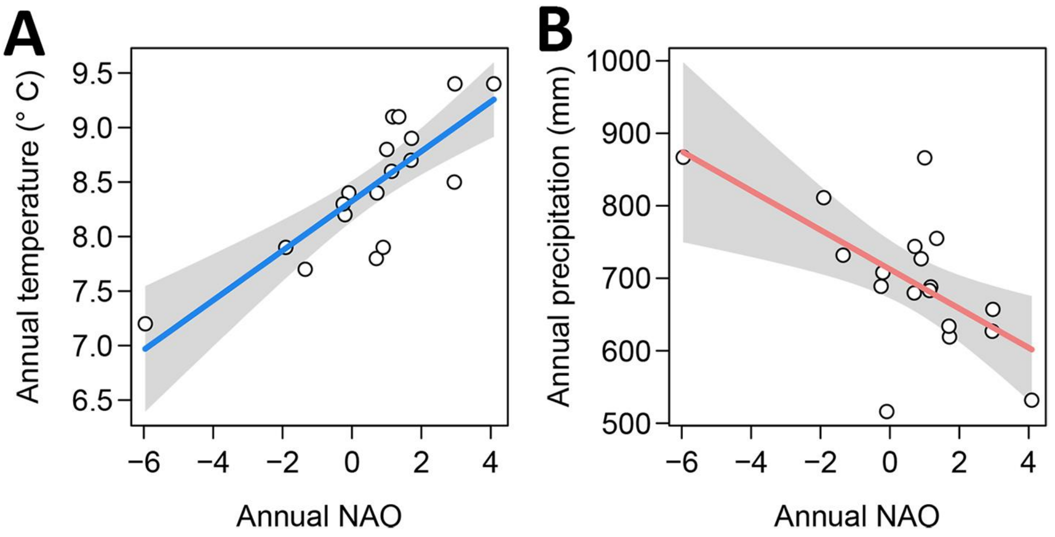

Appendix Figure 4. Annual NAO index in the Czech Republic is associated with a lower annual temperature mean (A) and higher annual sum of precipitation (B). The shaded areas indicated 95% confidence intervals.

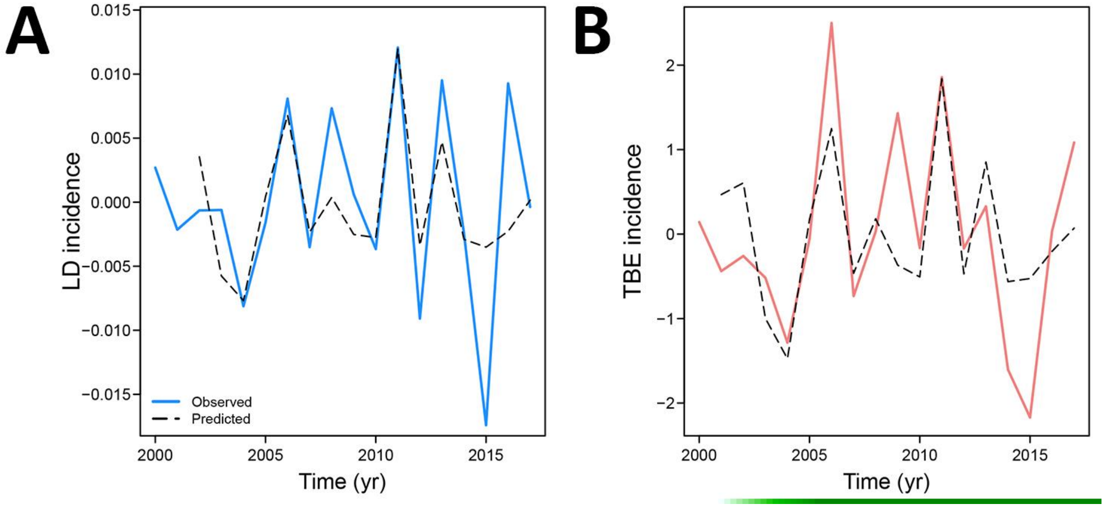

Appendix Figure 5. The observed (solid line) and predicted Lyme disease (LD) and tickborne encephalitis (TBE) incidences (dashed line) by best models with external predictors in the Czech Republic (A, B), Germany (C), Austria (D), and Slovenia (E). The fit is reasonably good even though the models are extremely simple containing just two predictors.

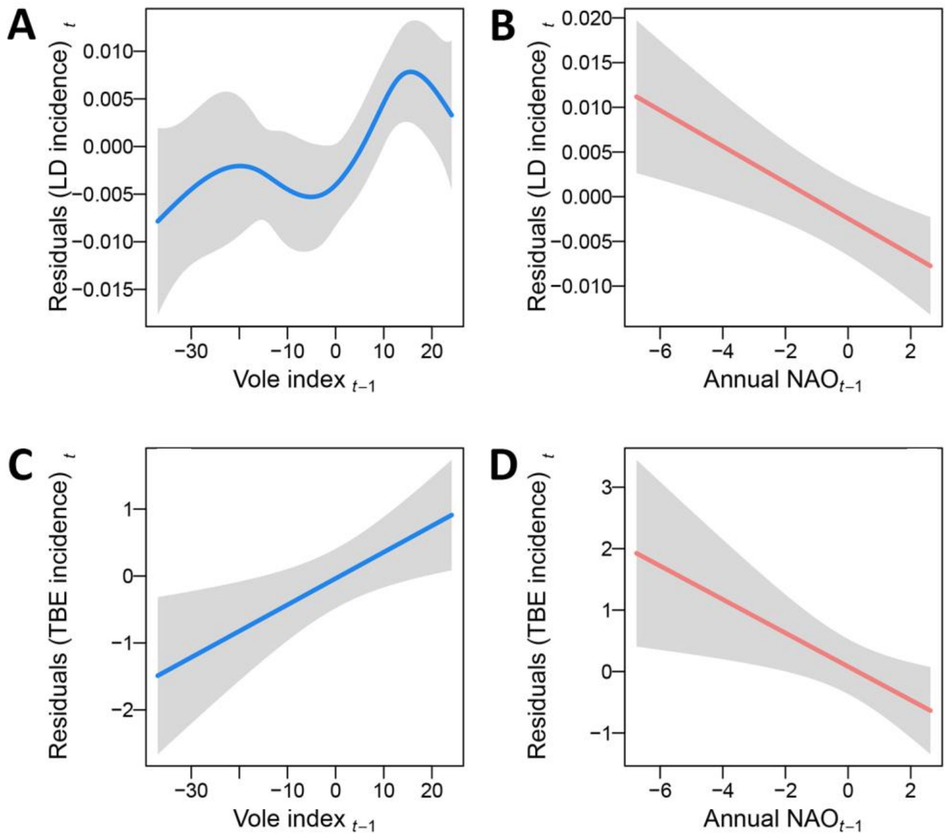

Appendix Figure 6. The effects of vole abundances (A, C) and annual NAO index (B, D) on Czech Lyme disease (LD) incidences (a, b) and Czech tickborne encephalitis (TBE) incidences (C, D) as visualized by generalized additive models (GAM) containing vole abundances and annual NAO index. GAM is a nonparametric regression which can accommodate nonlinearity in data. The y-axis shows the pure residual correlation after accounting for the variation caused by the second variable. The shaded areas show the 95% confidence intervals.

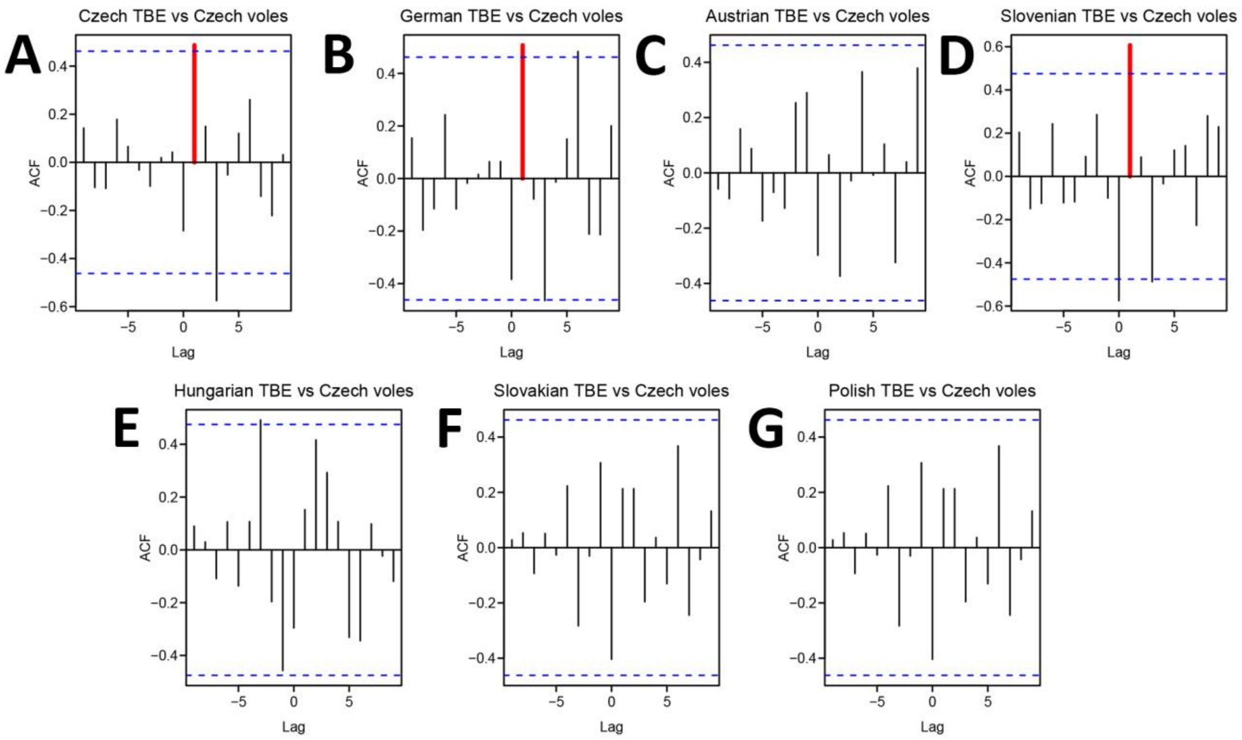

Appendix Figure 7. Cross-correlations between tickborne encephalitis (TBE) incidences from the Czech Republic (A), Germany (B), Austria (C), Slovenia (D), Hungary (E), Slovakia (F) and Poland (G) and Czech vole abundances. The dominant lag of 1 year is indicated in the Czech Republic (A), Germany (B) and Slovenia (C). A lag of 2 years is weakly indicated for Austria (C) and Hungary (E). In other countries, no clear pattern was observed. The meaningful lags are in red.

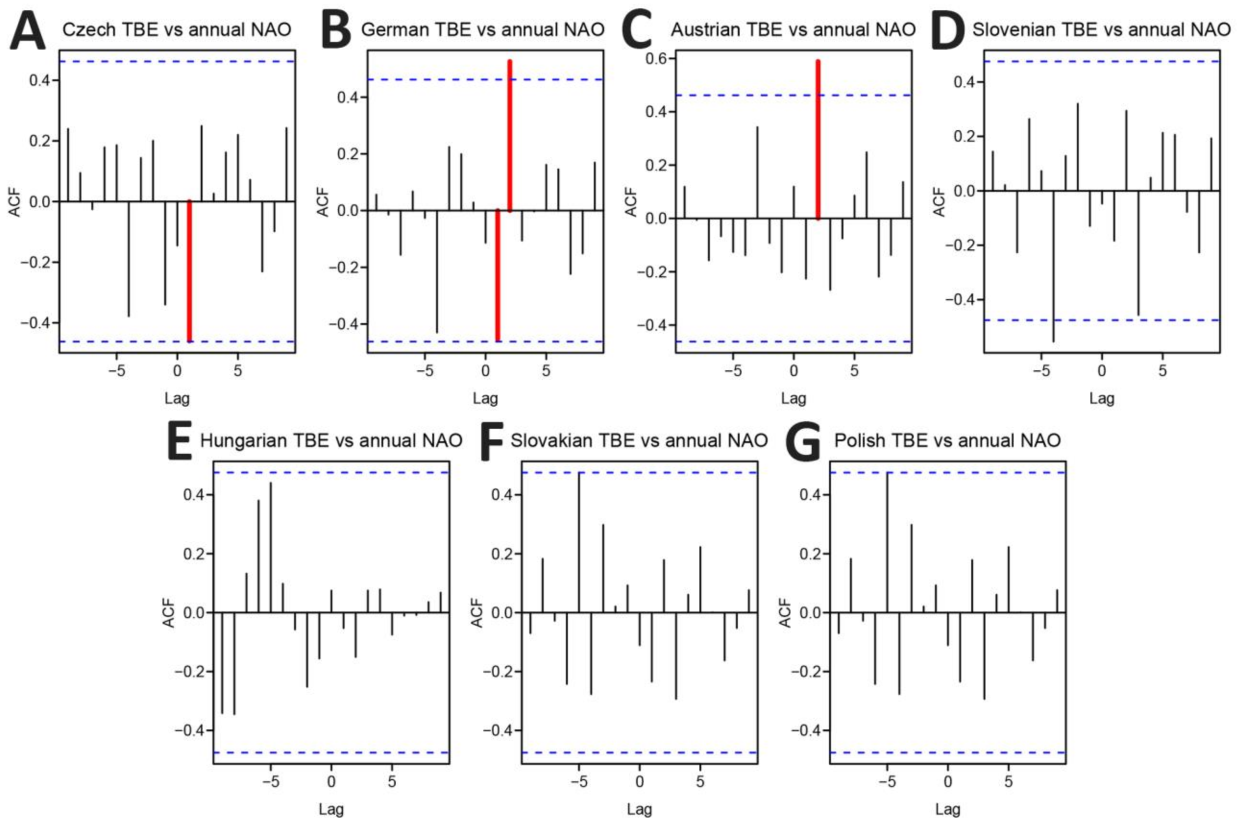

Appendix Figure 8. Cross-correlations between tickborne encephalitis (TBE) incidences from the Czech Republic (A), Germany (B), Austria (C), Slovenia (D), Hungary (E), Slovakia (F) and Poland (G) and annual NAO index. Time lag of 1 year is found for the Czech Republic (A), and Germany (B). A lag of 2 years strongly dominates in Germany (B), and Austria (C). The meaningful lags are in red.

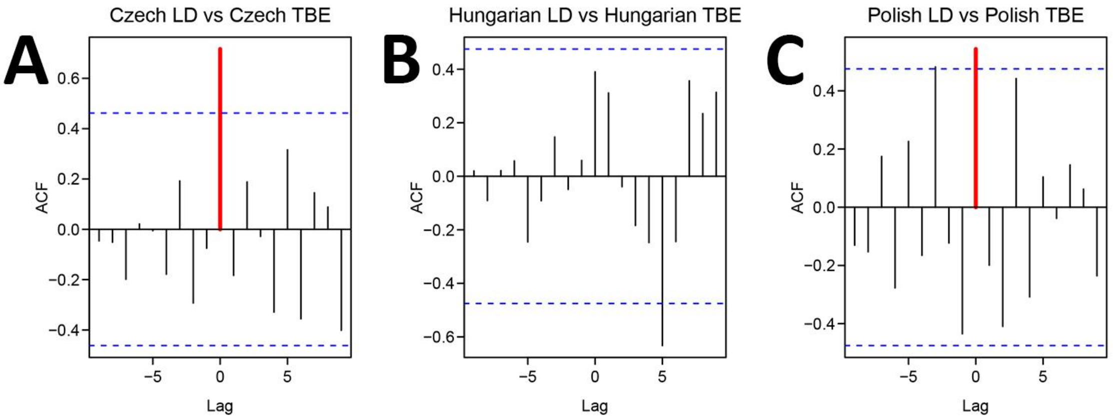

Appendix Figure 9. Cross-correlations between incidences of Lyme disease (LD) and tickborne encephalitis (TBE) in the Czech Republic (A), Hungary (B) and Poland (C). No lag is found for the studied countries. The meaningful lags are in red.
